# Diagnosis and Treatment of Synchronous Lymphoma and Digestive System Carcinoma: Report of Four Cases and Literature Review

**DOI:** 10.3389/fonc.2019.01367

**Published:** 2019-12-10

**Authors:** Jingshu Meng, Huaxiong Pan, Xiaoqian Li, Tao Liu, Zijian Liu, Qiuhui Li, Yin Xiao, Xinxiu Liu, Gang Wu, Fang Zhu, Liling Zhang

**Affiliations:** ^1^Cancer Center, Union Hospital, Tongji Medical College, Huazhong University of Science and Technology, Wuhan, China; ^2^Department of Pathology, Union Hospital, Tongji Medical College, Huazhong University of Science and Technology, Wuhan, China

**Keywords:** lymphoma, digestive system carcinoma, diagnosis, treatment, chemotherapy

## Abstract

**Objective:** To investigate the diagnosis and treatment of synchronous lymphoma and digestive system carcinoma and review literature.

**Materials and Methods:** We retrospectively analyzed the clinical data of four cases of synchronous lymphoma and digestive system carcinoma treated at our hospital. The clinical manifestations, pathological results, and treatment strategies were investigated.

**Results:** One of the four cases was diagnosed as follicular lymphoma with gastric adenocarcinoma, and the other three were diagnosed as diffuse large B-cell lymphoma with digestive system adenocarcinoma in the liver, sigmoid colon, and duodenum papilla, respectively. The second carcinoma was initially discovered incidentally because of the stage examination of lymphoma or the patient's poor response to treatment. The diagnosis of synchronous lymphoma and digestive system carcinoma depended mainly on the pathological examination.

**Conclusions:** The accurate diagnosis of synchronous malignancies is challenging because they rarely occur. We suggest a scrupulous re-biopsy of extranodal lesions in patients with lymphoma to improve the diagnostic accuracy of related double primary tumors. Age, performance status, symptoms, pathological types, and tumor staging should be considered when formulating a treatment strategy. The systemic treatment regimens should include drugs targeting the synchronous tumors in question, and these remain to be explored further.

## Introduction

Synchronous multiple primary cancers are defined as two or more tumors occurring within 6 months of each other. Synchronous lymphoma and digestive system adenocarcinoma are extremely rare. In our report, case 1 was diagnosed as follicular lymphoma (FL), and the other three cases were diffuse large B-cell lymphoma (DLBCL). FL is the second most common subtype of non-Hodgkin lymphoma (NHL) and the most widespread indolent lymphoma in western countries. The incidence rate of FL is 19–35% of all NHL in western countries but only about 5% in China (1). DLBCL represents the most common subtype of NHL and also the most recurrent aggressive lymphoma, accounting for 30–40% of all newly diagnosed lymphoma cases worldwide, with an even higher incidence in China (2).

We identified synchronous digestive system adenocarcinoma in our four lymphoma cases. The patients were diagnosed with lymphoma and synchronously with four different types of digestive system adenocarcinoma, namely, gastric adenocarcinoma, hepatocellular adenocarcinoma, sigmoid adenocarcinoma, and duodenal papilla adenocarcinoma. Among the listed digestive system tumors, the first three are widespread worldwide, but the fourth one is relatively rare. The incidence rate of primary duodenal papilla carcinoma is low, only accounting for 5% of gastrointestinal carcinoma and 0.01% of malignant tumors (3). Herein, we present four cases of synchronous lymphoma and digestive system adenocarcinoma, including the diagnosis and treatment of these rare conditions.

## Case Presentation

From January 2014 to January 2017, four patients diagnosed with synchronous lymphoma and digestive system malignancy were admitted to Cancer Center, Union Hospital, Tongji Medical College, Huazhong University of Science and Technology. The detailed characteristics of four patients were listed in Table 1.

**Table 1:** Clinical information for four synchronous digestive system adenocarcinoma and lymphoma patients.

**Case**	**Age (year)**	**Gender**	**Lymphoma**			**Digestive system carcinoma**			**Chemotherapy regimens**	**OS (months)**
			**Diagnosis**	**Staging**	**Treatment**	**Diagnosis**	**Staging**	**Treatment**		
1	61	Male	FL	IV	Chemo Radio	Gastric adenocarcinoma	-	Chemo, Ope	R-CHOP, R-GEMOX + S-1, R + S-1, Lenalidomide	53
2	58	Male	DLBCL	III	Chemo	Hepatocellular adenocarcinoma	I	Ope	CHOP	>62
3	78	Male	DLBCL	IV	Chemo	Duodenum papilla adenocarcinoma	–	Chemo	R-miniCHOP, S-1	16
4	65	Female	DLBCL	III	Chemo	Sigmoid colon adenocarcinoma	I	Chemo, Ope	R-CHOP, S-1	>33

*FL, follicular lymphoma; DLBCL, diffuse large B-cell lymphoma; Chemo, chemotherapy; Radio, radiotherapy; Ope, operation; OS, overall survival; R-CHOP, rituximab, cyclophosphamide, Adriamycin, vincristine, and prednisone; CHOP, cyclophosphamide, Adriamycin, vincristine, and prednisone*.

### Case 1

A 61-year-old male presented with multiple enlarged cervical lymph nodes, abdominal pain, and more than a month-long distension in February 2014. The patient had no personal or family medical history of a malignant neoplasm. A computed tomography (CT) scan revealed multiple enlarged lymph nodes. An excision biopsy of the left cervical enlarged lymph node showed FL grade 3. ^18^FDG positron emission tomography (PET)-CT confirmed that FDG-avid [standard uptake value (SUV)max 6.8] enlarged lymph nodes were located in the bilateral cervical, bilateral axillary, mediastinal, retroperitoneal, mesenteric, bilateral pelvic, and bilateral inguinal regions. PET-CT also showed focal pathological uptake in the angulus of the stomach (SUVmax 8.6), which was considered an infiltration of lymphoma into the stomach. Bone marrow biopsy was positive for lymphoma. The patient was diagnosed with stage IV FL (grade 3) with extranodal involvement of the stomach and bone marrow.

After two cycles of chemotherapy with R-CHOP (rituximab 375 mg/m^2^, day 1; cyclophosphamide 750 mg/m^2^, day 2; Adriamycin 50 mg/m^2^, day 2; vincristine 2 mg, day 2; prednisone 100 mg, days 2–6), an interim PET-CT showed that most of the initially high FDG uptake lesions disappeared, but residual FDG uptake (SUVmax 12.2) was found in the stomach, perigastric lymph nodes, and mesenteric lymph nodes. Gastroscopy and biopsy of gastric lesions were performed subsequently. Astonishingly, histopathology revealed the residues to be gastric tubular adenocarcinoma, not an infiltration of lymphoma to the stomach, as previously considered. The examination of the presence of *Helicobacter pylori* (Hp) was negative. Synchronous gastric cancer (GC) and FL were identified in this patient.

R-CHOP seemed to be ineffective on GC. Therefore, a regimen that could treat both GC and FL was needed. We continued chemotherapy with R-GEMOX plus Tegafur (S-1) (rituximab 375 mg/m^2^, day 1; gemcitabine 1,000 mg/m^2^, day 2; oxaliplatin 100 mg/m^2^, day 2; S-1 50 mg bid, days 4–17; treatment was repeated every 21 days). After two cycles of R-GEMOX plus S-1, PET-CT revealed that gastric lesions were resolved, but the perigastric and mesenteric lymph nodes still had high FDG uptake (SUVmax 7.2). At this point, the patient refused further treatment but returned 3 months later with disease progression. The patient's response after six cycles of R-GEMOX plus S-1 was partial remission (PR), with a residual lesion in the stomach and several enlarged intra-abdominal lymph nodes. For a second time, the patient refused to continue treatment after the seventh cycle of chemotherapy, and for the second time, he returned with disease progression 3 months later, with multiple lymph nodes in the retroperitoneal and bilateral pelvic regions.

Another two cycles of R-S1 (rituximab 375 mg/m^2^, day 1; S-1 50 mg bid, days 1–14; repeated every 21 days) and intensity-modulated radiation therapy were given, considering his performance status. The clinical target volume of radiotherapy included gastric lymph nodes in the perigastric, retroperitoneal, and bilateral pelvic regions. The response this time was complete remission (CR) after radiotherapy. Five months later, the patient was confirmed to have disease progression in multiple lymph nodes and the stomach again. Lenalidomide was added to the R-S1 regimen for two cycles. The lesions of the stomach were stable, and lymph nodes were shrinking. Then he underwent total gastrectomy in May 2017 because of the poor response to the chemotherapy of gastric cancer. Lenalidomide was used as a maintenance treatment to control lymphoma after the operation. However, the patient died of respiratory and circulatory failure associated with tumor progression in August 2018 with the survival time of 53 months.

### Case 2

In May 2014, a 58-year-old male was admitted to our hospital for the evaluation of an abnormal lesion in the right liver lobe revealed by an upper abdominal CT scan taken at a routine health check a few days before. He had a history of hepatitis B virus infection but no personal or family medical history of a malignant neoplasm. Surgical hepatolobectomy was performed, and following histopathology revealed a well-differentiated hepatocellular carcinoma (HCC) in Couinaud's segments 5 and DLBCL originating from activated B cell (non-GCB subtype) in synchronous perihepatic lymph nodes. A CT scan exposed multiple enlarged lymph nodes in the bilateral cervical, right axillary, mediastinal, hilar, retroperitoneal, and bilateral inguinal regions. A bone marrow biopsy was negative for lymphoma. Finally, the patient was diagnosed with synchronous stage III DLBCL, non-GCB subtype, and stage I HCC. Because the patient could not afford treatment with rituximab, he received eight cycles of chemotherapy with CHOP. The response to the therapy was CR. As of the write-up of this manuscript, the patient still lives a good quality of life without tumor progression till now and has already survived for more than 62 months.

### Case 3

A 78-year-old male was admitted to our hospital with a right inguinal mass in July 2014. An abdominal CT scan revealed multiple enlarged lymph nodes at the para-aortic, bilateral iliac, and right inguinal regions. Besides a lesion at the distal end of the choledoch, intrahepatic and extrahepatic bile duct dilation was also detected. The patient had no personal or family medical history of a malignant neoplasm. Aspiration biopsies on the right inguinal enlarged lymph nodes were performed, and histopathology showed DLBCL originating from germinal center B cell (GCB subtype). A further PET-CT confirmed abnormal FDG uptake (SUVmax 15.9) in the retroperitoneal, mesenteric, bilateral common iliac, right internal and external iliac, and right inguinal lymphatic regions, as well as in the bilateral testes. Bone marrow biopsy was positive for lymphoma. The patient was diagnosed with a stage IV DLBCL, GCB subtype, involving the bone marrow. Since PET-CT revealed no abnormal FDG uptake in the ampullary area, we considered the lesion shown in the CT images to be duodenal papilla or a benign lesion that could be under observation temporarily. Subsequently, treatment for lymphoma was performed.

After four cycles of R-miniCHOP (rituximab 375 mg/m^2^, day 1; cyclophosphamide 400 mg/m^2^, day 2; epirubicin 37.5 mg/m^2^, day 2; vincristine 1 mg, day 2; prednisone 60 mg, days 2–6), an interim PET-CT was performed, and it revealed significant inhibition of lesions' metabolic activity (SUVmax 2.7). The response was PR. However, further chemotherapy could not be continued due to the gradually rising amounts of alanine aminotransferase (ALT) (up to 362 U/L), aspartate aminotransferase (AST) (up to 395 U/L), total bilirubin (up to 164.8 μmol/L), and direct bilirubin (up to 85.8 μmol/L) during treatment. We identified and attributed jaundice to impaired bile flow possibly due to the lesions located in the ampulla of the Vater found in previous CT scan images. An endoscopic ultrasound-guided fine-needle aspiration biopsy was, hence, performed immediately. At the same time, an endoscopic retrograde biliary stent placement drainage was performed to relieve the biliary obstruction. Unfortunately, histopathology confirmed duodenum papilla adenocarcinoma. The examination of the presence of Hp was negative. Synchronous GCB-DLBCL and duodenum papilla adenocarcinoma were diagnosed in this patient.

Given the patient's age and poor performance status, surgery for duodenum papilla adenocarcinoma was not recommended. He also refused to undergo chemotherapy. Five months later, he presented with abdominal pain and distension again. Abdominal CT scan showed papilla adenocarcinoma under progression. With the patient's consent, S-1 (50 mg bid, days 1–14; repeated every 21 days) was used to control papilla adenocarcinoma. But the therapy was ineffective, and he died because of tumor progression with the survival time of 16 months.

### Case 4

A 65-year-old female was referred to our hospital in October 2016 with multiple cervical and supraclavicular lymph node enlargement for nearly 2 years. The patient had no personal or family medical history of a malignant neoplasm. Excision of the right enlarged cervical lymph node was performed, and histopathology showed a non-GCB subtype DLBCL. Further PET-CT confirmed multiple FDG-avid (SUVmax 49.2) enlarged lymph nodes located in the right parapharyngeal, right cervical, right clavicular, right axillary, lesser stomach curvature, para-pancreatic, and retroperitoneal regions, which matched the signs of lymphoma infiltration. The result of a bone marrow biopsy was normal. PET-CT also showed a focal pathological uptake in the sigmoid colon region (SUVmax 9.2), and enteroscopy was recommended to exclude other diseases. Colonoscopy was performed, and an ulcerative lesion was found. Histopathology demonstrated adenocarcinoma of the sigmoid colon, not lymphoma involving the colon. In all, the patient was diagnosed with synchronous stage III non-GCB subtype DLBCL and sigmoid colon adenocarcinoma.

After obtaining her informed consent, R-CHOP chemotherapy treating lymphoma was given first. During the interval of chemotherapy, neoadjuvant chemotherapy with S-1 (50 mg bid, days 1–14; repeated every 21 days) was administered to treat sigmoid colon adenocarcinoma. After two cycles of R-CHOP, a CT scan was performed and showed that the response was CR. After three cycles of R-CHOP and two cycles of S-1, laparoscopic radical sigmoidectomy was performed. Postoperative pathologic staging was pT2N0M0, stage I. Postoperative adjuvant chemotherapy was not necessary. Then she continued to receive treatment and completed six cycles of R-CHOP and two cycles of S-1. She has been living free of tumor recurrence for nearly 33 months now.

## Discussion

The gastrointestinal tract is the most common organ for the extranodal involvement of lymphoma. However, the coexistence of digestive system adenocarcinoma and lymphoma is extremely rare. The patient in case 1 was the second reported case of synchronous FL and GC. The clinical characteristics of these two cases are reviewed in Table 2 (4). In case 2, the patient was the fifth reported case of synchronous DLBCL and HCC, the clinical characteristics of these five cases are reviewed in Table 3 (5–8). Synchronous intestinal carcinoma and lymphoma were diagnosed in cases 3 and 4; relevant reported instances are listed in Table 4 (9–11).

**Table 2:** Clinical characteristics of reported cases of synchronous gastric carcinoma and FL.

**References**	**Age (year)**	**Gender**	**Lymphoma**			**Gastric carcinoma**			**Chemotherapy regimens**	**OS (months)**
			**Diagnosis**	**Staging**	**Treatment**	**Diagnosis**	**Staging**	**Treatment**		
Xue et al. (4)	68	Female	FL	IVB	Chemo	Gastric moderately differentiated adenocarcinoma	Not described	Chemo	R-CHOP, 5-FU + L-OHP + VP-16	Not described
Present case	61	Male	FL (grade 3)	IV	Chemo Radio	Gastric tubular adenocarcinoma	-	Chemo, Ope	R-CHOP, R-GEMOX + S-1, R + S-1, Lenalidomide	53

*FL, follicular lymphoma; 5-FU, 5-fluorouracil; L-OHP, oxaliplatin; VP-16, etoposide; VCR, vincristine; DXM, dexamethasone; OS, overall survival*.

**Table 3:** Clinical characteristics of reported cases of synchronous hepatocellular carcinoma (HCC) and lymphoma in the literature.

**References**	**Age (year)**	**Gender**	**Lymphoma**			**Hepatocellular carcinoma**				**Chemotherapy regimens**	**OS (months)**
			**Diagnosis**	**Staging**	**Treatment**	**Diagnosis**	**Number**	**Hepatitis virus infection**	**Treatment**		
Suriawinata et al. (5)	55	Male	DLBCL	Not described	–	Well-differentiated HCC	2	HCV	Liver transplantation	Not described	>15
Kataoka et al. (6)	64	Male	DLBCL	Not described	Conservative therapy	Well-differentiated	1	HCV	Conservative therapy	–	1.5
Lin et al. (7)	70	Male	DLBCL	Not described	Chemo	Necrotic tumor tissue with few HCC cells	Multiple	HCV	Not described	R-CHOP	NA
Lee et al. (8)	60	Male	FL	Not described	Chemo	HCC	2	HCV	TACE and RFA	R-CVP	>20
Present case	58	Male	DLBCL	III	Chemo	Well-differentiated	1	HBV	Hepatolobectomy	CHOP	>62

*FL, follicular lymphoma; TACE, transcatheter arterial chemoembolization; RFA, radiofrequency ablation; DLBCL, diffuse large B-cell lymphoma; Chemo, chemotherapy; Ope, operation; OS, overall survival; R-CHOP, rituximab, cyclophosphamide, Adriamycin, vincristine, and prednisone; R-CVP, rituximab, cyclophosphamide, vincristine, and prednisone; HBV, hepatitis B virus; HCV, hepatitis C virus*.

**Table 4:** Clinical characteristics of reported cases of synchronous intestinal carcinoma and lymphoma in the literature.

**References**	**Age (year)**	**Gender**	**Lymphoma**			**Hepatocellular carcinoma**			**Chemotherapy regimens**	**OS (months)**
			**Diagnosis**	**Staging**	**Treatment**	**Diagnosis**	**Staging**	**Treatment**		
Kidd et al. (9)	67	Female	HL	IIA	Chemo	Rectal moderately differentiated adenocarcinoma	pT2 N0 M0, Dukes' A	Ope	ABVD	>27
	74	Female	DLBCL	Not described	–	Cecal adenocarcinoma	pT3 N0 M0, Dukes' B	–	–	<1
	65	Male	HL	III	Chemo	Rectal moderately differentiated adenocarcinoma	ypT3 yN2 M0, Dukes' C1	Ope, Chemo	ABVD, capecitabine	>12
	62	Female	HL	I	watch and wait	Sigmoid colon moderately differentiated adenocarcinoma	pT3 N0 M0, Dukes' B	Ope	–	>29
	33	Male	FL (grade 1)	I	-	Rectosigmoid moderately differentiated adenocarcinoma	pT3 N0 M0, Dukes' B	Ope	–	>120
Cui et al. (10)	53	Female	DLBCL	Not described	Chemo	Sigmoid colon adenocarcinoma	pT3N0M0, stage II	Ope	R-CHOP	7
Yang et al. (11)	73	Male	DLBCL	II	Chemo	Sigmoid colon well-differentiated adenocarcinoma	pT3N0M0, stage IIA	Ope	R-CHOP	>4
Present case	78	Male	DLBCL	IV	Chemo	Duodenum papilla adenocarcinoma	–	Chemo	R-miniCHOP, S-1	16
	65	Female	DLBCL	III	Chemo	Sigmoid colon adenocarcinoma	pT2 N0 M0, I	Chemo, Ope	R-CHOP, S-1	>33

*ABVD, Adriamycin, bleomycin, vincristine, dakabazine; DLBCL, diffuse large B-cell lymphoma; Chemo, chemotherapy; Ope, operation; R-CHOP, rituximab, cyclophosphamide, Adriamycin, vincristine, and prednisone; OS, overall survival; FL, follicular lymphoma; HL, Hodgkin lymphoma*.

Since it is a rare phenomenon, the diagnosis of synchronous multiple primary cancer is difficult. Although it is unnecessary and infeasible to obtain a biopsy from every suspicious lesion, the awareness of the different biological behaviors between the digestive system tumor and lymphoma should be considered critical to diagnosis and treatment. In cases 1 and 4, diagnosed with lymphoma first, certain gastrointestinal high metabolic regions revealed by an interim PET-CT remained unchanged, appearing to defy the habitual response to chemotherapy. In case 3, the symptom of bile duct obstruction occurred while abnormal metabolic lymph node regions shrunk. These clinical observations were worth paying attention to for the possibility of primary synchronous digestive system carcinoma. And the accuracy of diagnosis was made based on the re-biopsy.

A PET-CT scan was recommended recently for initial staging, identifying extranodal lesion and response assessment in both FL and DLBCL, which is more accurate and sensitive than contrast-enhanced CT scanning (12–15). However, false-positive results with a PET-CT in FL have been described and occurred with inflammatory, normal physiologic processes, additional malignancy, and benign lesions. A retrospective study by Iwamuro et al. (16) found that the true-positive rate of ^18^F-FDG uptake in the gastrointestinal lesions was only 46.3% in 41 patients with gastrointestinal lesions of FL. In contrast, the false-negative rate was 58.5%, whereas the false-positive rate was 12.2%. So, when patients with FL have FDG uptake in the gastrointestinal tract, endoscopic examination and biopsy should be performed to confirm the diagnosis of gastrointestinal lesions (17). Compared to indolent lymphoma, the accuracy of PET in the evaluation of gastrointestinal involvement with aggressive lymphoma is higher. Kumar et al. (18) reported that the sensitivity, specificity, positive, and negative predictive values of PET for aggressive gastrointestinal lymphoma were 86, 100, 100, and 92%, respectively. Yi et al. (19) reported that the mean SUV of gastric DLBCL was 15.22. Radan et al. (20) revealed that the SUVmax of aggressive gastric lymphoma was 19.7 ± 11.5, whereas that of mucosa-associated lymphoid tissue (MALT) lymphoma was only 5.4 ± 11.5. From these data, we can conclude that the SUVmax of the gastrointestinal lesion was at a high level in aggressive lymphoma, especially in DLBCL. In cases 3 and 4 of our research, SUVmax was 15.9 and 49.2 accordingly with higher SUV in lymphoma. In case 3, there was no abnormally uptaken radioactivity in the ampullary area. So, when the SUVmax or the response to chemotherapy of the gastrointestinal lesion is different from those of other lesions, other diseases should be considered, and further biopsy should be performed.

There is no standard treatment strategy for synchronous lymphoma and digestive system carcinoma. Individual treatments should be based on the different therapeutic principles of the synchronous tumors, combined with the doctor's experience and close attention to the characteristics of the patient. FL represents the majority of indolent NHL, and more than 70% of patients have an overall survival (OS) longer than 10 years, especially with current therapies. But the prognosis of advanced-stage GC is weaker than that of FL. Namikawa et al. (21) reviewed 57 patients diagnosed with synchronous GC and lymphoma and found that the prognosis of patients with synchronous GC and lymphoma might depend more on GC than on lymphoma, in which case the treatment and therapeutic outcomes could depend on the adenocarcinoma status. Chen et al. (22) reported the case of a patient with synchronous early-stage GC and lymphoma, who was treated successfully by combining laparoscopic resection and adjuvant chemotherapy. Cammarota et al. (23) reported a patient with synchronous GC and MALT lymphoma who was successfully treated with neoadjuvant chemotherapy and total gastrectomy. Following the approaches of previous findings, treatment strategies in case 1 were mainly determined based on GC rather than FL.

In case 1, the patient was diagnosed with synchronous GC and FL because of the different responses to chemotherapy. Since most of grade 3 FL is sensitive to immunochemotherapy, we presumed that these lymph nodes resistant to treatment might not be an outcome of FL infiltration but that of GC metastasis. GC with lymph node metastasis is inoperable. Treatment for advanced-stage GC includes chemotherapy, radiotherapy, and target therapy. For patients with synchronous GC and FL, systemic therapy regimens must consist of drugs that can impart sensitivity to two different kinds of tumors. Gemcitabine plus oxaliplatin in combination with folinic acid and infusional 5-fluorouracil (5-FU) (Gem-FOLFOX) has shown significant antitumor activity in advanced GC patients (24). S-1, a prodrug of 5-FU, is one of the main anticancer drugs used worldwide in gastrointestinal cancers (25). The combination of S-1 plus cisplatin is also considered active and tolerable in GC patients (26). On the other hand, rituximab plus gemcitabine and oxaliplatin (R-GEMOX) is highly effective in NHL (27). So, when the patient was diagnosed with synchronous GC and FL, we used R-GEMOX plus the S-1 regimen, which was effective and tolerable.

As for DLBCL, it typically presents as an aggressive lymphoma, evolving over months and resulting in a symptomatic disease that would be fatal without treatment. Thus, the therapy for DLBCL should be administered immediately once diagnosed not only because of its aggressive behavior but also because of the high remission rate after chemotherapy (R-CHOP) in most patients, even in advanced cases. When lymphoma is controlled, the effective treatment of the digestive system carcinoma should be carried out per patient's condition. Surgery plays a vital role in early-stage HCC and gastrointestinal carcinoma. In case 2, the final diagnosis of HCC and DLBCL was confirmed by pathology simultaneously. The patient suffered from early-stage HCC and advanced-stage DLBCL. Therefore, postoperative adjuvant therapy for HCC was not necessary, and only R-CHOP was adopted for DLBCL. In case 3, the patient presented with obstructive jaundice after four cycles of R-miniCHOP, causing doubts as to the existence of another disease. The endoscopic ultrasound-guided fine-needle aspiration biopsy revealed adenocarcinoma of the duodenal papilla. Standard pancreaticoduodenectomy usually is the primary treatment for duodenal papilla carcinoma (28–30). However, considering the patient's age and poor performance status, a palliative endoscopic retrograde biliary stent placement drainage was performed to relieve his biliary obstruction. While clinical randomized controlled trials and proof from evidence-based medicine are insufficient in the chemotherapy of duodenal papilla carcinoma, systemic chemotherapy with fluoropyrimidine 5-FU derivatives and their combinations with other agents have been revealed to be effective in duodenal adenocarcinoma (31–33). Katakura et al. reported that S-1 treatment of duodenal cancer was highly efficient, with a survival time of 19 months (34). Oral S-1 was also once reported to be successfully used in the treatment of ampullary carcinoma in an old patient (35). So, we chose S-1 to treat duodenal papilla adenocarcinoma. In case 4, lymphoma and sigmoid carcinoma were both diagnosed before treatment. Given the patient's decent condition and no evidence of sigmoid carcinoma metastasis, surgery for removing sigmoid carcinoma was recommended. S-1, used as monotherapy or in combination with chemotherapy, has been demonstrated to be an appropriate alternative treatment of metastatic colon cancer, as well as stage III postoperative colon cancer (36–38). So, we chose S-1 as the neoadjuvant chemotherapy to control colon carcinoma growth and metastasis during the interval of chemotherapy for lymphoma mainly for its low toxicity and acceptable tolerance.

In summary, these cases highlight the difficulty in accurately diagnosing synchronous multiple primary cancers, given that they rarely occur. Herein, we suggest a scrupulous biopsy of the extranodal lesion in patients with lymphoma to improve the diagnostic accuracy of the related double primary tumors. The standard treatment strategy remains unclear. Age, performance status, symptoms, pathological types, and tumor staging should be considered crucial when formulating a treatment strategy. The systemic treatment regimens should include drugs targeting the synchronous tumors in question.

## Data Availability Statement

The datasets generated for this study are available on request to the corresponding author.

## Ethics Statement

The studies involving human participants were reviewed and approved by Tongji Medical College, Huazhong University of Science and Technology. The patients/participants provided their written informed consent to participate in this study. Written informed consent was obtained from the individual(s) for the publication of any potentially identifiable images or data included in this article.

## Author Contributions

JM and HP contributed equally to this article. FZ and JM wrote the manuscript. HP and QL collected the data. XLi and ZL analyzed the data. YX and XLiu revised the manuscript. GW and TL designed the manuscript. LZ reviewed the manuscript.

### Conflict of Interest

The authors declare that the research was conducted in the absence of any commercial or financial relationships that could be construed as a potential conflict of interest.
